# Unveiling mitochondrial and PANoptosis-related biomarkers for premature ovarian insufficiency

**DOI:** 10.1186/s13048-025-01839-4

**Published:** 2025-12-22

**Authors:** Zhen Ma, Hong Sun

**Affiliations:** Gynecology West Hospital District, YiDu Central Hospital of Weifang, Shandong Second Medical University, Qingzhou City, Shandong Province 262500 China

**Keywords:** Premature ovarian insufficiency, PANoptosis, Mitochondria, Biomarker

## Abstract

**Background:**

Advances in diagnosis and treatment of premature ovarian insufficiency (POI) are urgently needed. Mitochondrial dysfunction and PANoptosis are associated with ovarian damage. In this study, we aimed to identify mitochondrial and PANoptosis-related biomarkers for POI.

**Methods:**

Integrative bioinformatics analysis was performed based on RNA sequencing datasets from the Gene Expression Omnibus (GEO) database, combined with the MitoCarta3.0 database and PANoptosis-related genes. The feature differentially expressed genes (DEGs) were screened using machine learning methods. Based on the expression levels of mitochondrial characteristic genes (ACSM3, ALDH1L1, MAOB, OSBPL1A) and pan-apoptotic genes (NOS2, UCHL1) screened out by the above machine learning, the mitochondrial and PANoptosis phenotypic scores were calculated through the GSVA algorithm. Differential expression of biomarkers was validated in clinical samples, and the role of nitric oxide synthase 2 (*NOS2*) was explored in POI mouse models.

**Results:**

Four mitochondrial-related genes (*ACSM3*, *ALDH1L1*, *MAOB*, and *OSBPL1A*) and two PANoptosis-related genes (*NOS2* and *UCHL1*) were screened. The mitochondrial phenotype scores of the samples were positively correlated with PANoptosis scores. All biomarkers were downregulated in human granulosa cells. Notably, *NOS2*, identified as the most significant biomarker, was downregulated in the POI model. Moreover, the overexpression of *NOS2* markedly suppressed cell death and reactive oxygen species (ROS) production in the ovarian tissues of POI mice.

**Conclusions:**

The identified biomarkers may play key roles in the pathology of POI and serve as candidate biomarkers for its diagnosis and treatment.

**Supplementary Information:**

The online version contains supplementary material available at 10.1186/s13048-025-01839-4.

## Background

Premature ovarian insufficiency (POI), also known as premature ovarian failure, affects at least 1% of women of reproductive age [1]. POI is characterized by decreased ovarian function and reproductive hormone deficiency in women under 40 years old [2]. This medical condition is associated with subfertility or infertility and increases the risk of osteoporosis, cardiovascular disease, and emotional challenges [3]. POI has widespread effects on women’s health and quality of life. Currently, treatments are limited because of their incomplete effectiveness and severe side effects [4, 5]. Advances in diagnosis and management are imperative to address both the physical and psychological impacts of this condition.

Recent evidence has shown that mitochondrial dysfunction is associated with the pathogenesis of POI [6]. Disruption of mitochondrial function leads to decreased oocyte quality and a potential reduction in the fertilization process and embryo development [7]. Mitochondrial dysfunction-induced reactive oxygen species (ROS) accumulation, is associated with increased oxidative stress, leading to inflammation, apoptosis, and necrosis of germ cells in the ovaries [8]. Mitochondrial tRNA gene mutations result in decreased ATP production and ROS accumulation, which play active roles in the progression of POI [9]. Genetic disorders of POLG are associated with oxidative stress and mitochondrial dysfunction in POI [10]. Manipulation of mitochondrial function may offer new insights into the diagnosis and treatment of POI.

PANoptosis is a specific type of inflammatory programmed cell death controlled by the PANoptosome complex. It is characterized by integrated features of cellular pyroptosis, apoptosis, and/or necroptosis [11]. PANoptosome is a lethal protein complex that drives pyroptosis, apoptosis and necrosis [12]. At present, PANoptosis plays a role in many human infections, autoimmune diseases and cancers [11–14]. Recent evidence suggests that cellular PANoptosis is elevated in mice with ovarian damage [15]. Cranberry-derived exosomes relieve murine POI by reducing the mRNA and protein expression of PANoptosis-related factors [16]. Thus, we speculated that PANoptosis plays a key role in the pathogenesis of POI.

Mitochondrial dysfunction directly triggers the decline in oocyte quality through insufficient ATP production and ROS accumulation. PANoptosis accelerates the depletion of ovarian reserve by integrating the pyroptosis/apoptosis/necrosis pathway. Therefore, we speculate that there is a synergistic effect between the two in the pathogenesis of POI. Considering the critical roles of mitochondria and PANoptosis in the progression of POI, we focused on mitochondrial and PANoptosis-related genes. POI-related datasets (GSE201276 and GSE245155) from the Gene Expression Omnibus (GEO) database were combined with MitoCarta3.0 and PANoptosis-related gene sets. Differentially expressed genes (DEGs) related to mitochondria and PANoptosis in POI were analyzed. The featured genes were filtered using machine learning methods. We attempted to explore the candidate biomarkers to open the new window for the diagnosis and treatment of POI.

## Methods

### Gene expression profiles acquisition

The gene expression profiles of POI (accession numbers: GSE201276 and GSE245155) were downloaded from the GEO database [17]. The GSE201276 dataset included 11 samples from six patients with POI and five control patients. GSE245155 contained ten primordial germ cell samples from eight patients with POI and two controls. In this study, GSE201276 was used as the training dataset and GSE245155 as the validation dataset.

### Mitochondrial and PANoptosis-related genes download

Mitochondria-related genes were retrieved from the human MitoCarta3.0 database [18]. PANoptosis is a type of cell death characterized by apoptosis, pyroptosis, and necroptosis. Apoptosis-related genes were obtained from a study by Zhou et al. [19]. Pyroptosis- and necroptosis-related genes were derived from previous studies by Qin et al. and Song et al. [20, 21]. After de-duplication, 1765 genes were obtained as the PANoptosis gene set for subsequent analysis.

### Differentially expressed gene analysis

DEGs were analyzed using the GSE201276 dataset. The DEGs between POI and the control samples were identified using the Limma software package [22], with *P*-values < 0.05, and |logFC|>0.585 [23].

### Mitochondrial and PANoptosis-related DEGs analysis and function enrichment analysis

The differential expression of mitochondrial- and PANoptosis-related genes was analyzed using a Venn diagram-based approach. DEGs were intersected with mitochondrial- and PANoptosis-related genes. Subsequently, the intersections were subjected to GO enrichment analysis using ‘clusterProfiler’ R package [24] (version 4.7.1, http://bioconductor.org/packages/release/bioc/html/clusterProfiler.html).

### Machine learning algorithms

POI-related biomarker genes were screened using two machine learning methods. A LASSO regression model was constructed using the glmnet package [25] (version 4.1–6.1, httRiskscore://cran.r-project.org/web/packages/glmnet/index.html). The optimal Lasso λ value was selected via ten-fold cross-validation. Feature genes with the minimum squared errors were filtered. Boruta is an extension of the regular random forest method, which improves the accuracy, stability, and reliability of feature selection [26]. In the present study, the Boruta model was constructed using the Boruta R package [25] (version 8.0.0; https://cran.r-project.org/web/packages/Boruta/index.html). Boruta generates shuffled copies of all real features as shadow features and trains a random forest classifier to determine the importance of the features. Finally, biomarker genes were identified by intersecting the feature genes selected by both machine learning methods. Differential expression of these biomarker genes was determined in the training and validation datasets.

### Nomogram model construction

To dissect the specific role of biomarker genes in predicting the risk of POI, R 4.21 package rms version 5.1-2.1.1 was used to construct a nomogram model based on the expression values of biomarker genes. The points of each biomarker gene in each sample were calculated based on the regression coefficient. The total points for each sample were calculated and the probability of the outcome for each sample was determined using a transformation function.

### Phenotypic scores of mitochondria and PANoptosis

Based on the expression levels of 1,136 mitochondrial-related genes and 1,765 pan-apoptosis-related genes screened from the above analysis, the mitochondrial and PANoptosis phenotypic scores were calculated using the GSVA algorithm. Spearman correlation analysis was performed to assess the relationship between these two phenotypic scores in normal and POI samples. The results were visualized using correlation scatter plots.

### Transcriptional regulation analysis of biomarker genes

Gene expression is regulated by transcription factors (TFs) and microRNAs (miRNAs). To explore the regulatory mechanisms of biomarker genes, TFs were predicted based on the human Transcription factor database (http://bioinfo.life.hust.edu.cn/hTFtarget#!/). The putative binding sites of miRNAs on the biomarkers were predicted using miRWalk3.0. The gene regulatory network was developed using Cytoscape software (version 3.9.2).

### Prediction of small molecule drugs

The drug-gene interaction database (DGIdb 5.0) is a collection of drug-gene interactions [27]. Clinically relevant drug-gene interactions for the identified biomarkers were mined from this database.

### Clinical samples

From January 2024 to March 2025, eight pairs of granulosa cell samples were obtained from eight patients with POI and healthy controls with informed consent. The samples were obtained during surgical procedures, such as in vitro fertilization or embryo transfer. Patients aged less than 40 years with a history of four months of oligo- or amenorrhea were included in the study. The baseline clinical metadata of the two participant groups are presented in Table 1. The results demonstrate that the two groups were comparable in demographic characteristics (age and BMI), while the differences in clinical features (AMH, FSH, E2, number of oocytes retrieved) aligned with the pathophysiological manifestations of POI, supporting the internal validity of subsequent studies. Approval was obtained from the Ethics Committee of our hospital, and all procedures were conducted in accordance with the Helsinki guidelines.

**Table 1:** Basic clinical metadata of the two groups of participants

‌Parameter‌	‌POI group (*n* = 8)‌	‌Control group (*n* = 8)‌	‌P-value‌
Age (years)	35.25 ± 3.41	34.13 ± 3.48	0.525
BMI (kg/m²)	21.66 ± 1.52	21.80 ± 1.49	0.849
AMH (ng/mL)	0.56 ± 0.19	3.12 ± 0.77	< 0.001
FSH (IU/L)	39.87 ± 5.78	6.86 ± 1.03	< 0.001
E2 (pg/mL)	19.99 ± 1.52	79.78 ± 9.50	< 0.001
Number of oocytes retrieved	2.13 ± 0.99	9.50 ± 1.41	< 0.001

Data presented as mean ± SD. *P* < 0.05 indicates significant difference

### POI animal model construction

Animal experiments were approved by the Institutional Animal Care and Use Committee and were performed according to the animal care and use guidelines. Healthy six-week-old adult female C57BL/6J mice were obtained from the National Laboratory Animal Center. The animals were acclimatized in cages under standard laboratory conditions (12 h/12 h light/dark cycles) for one week with free access to food and water. To simulate ovarian failure in vivo, a chemotherapy-induced POI model was constructed. A single intraperitoneal injection of 90 mg/kg cyclophosphamide (CTX) was administered to induce the POI model, while the control group received an equivalent volume of normal saline. Following CTX treatment, mice underwent a 14-day recovery period to establish stable ovarian dysfunction. Vaginal exfoliated cell smears are collected every morning at 8 o’clock for two consecutive weeks. An irregular estrous cycle confirms successful model establishment. Compared to the control group, the model group exhibited irregular cycles, characterized by persistent diestrus or alternating diestrus and metestrus, with infrequent estrus and proestrus stages. The control group displayed typical estrous cycles.

### Nitric oxide synthase 2 (NOS2) lentiviral vector construction and packaging

The recombinant lentiviral vector carrying the *NOS2* gene sequence (LV-NOS2) and the negative control (LV-NC, empty vector) were constructed and purchased from GeneChem Biotechnology (Shanghai, China). LV-NOS2 or LV-NC, together with packing vectors, were co-transfected into HEK293T cells for packaging. Finally, the vectors were condensed to a titer of 2 × 10^8^ transducing units (TU)/mL. After successful modeling, the mice were anesthetized with 2% isoflurane, and a 5 µL intra-ovarian injection of LV-NOS2/LV-NC (2 × 10⁸ TU/mL) or PBS was administered. ‌Only the right ovary was injected‌ under sterile conditions.

### Treatment and grouping

All the mice were randomly divided into four groups (*n* = 6 per group): control group (no treatment), LV-NOS2 group (mice received a 5 µL intra-ovarian microinjection of LV-NOS2 at a titer of 2 × 10^8^ TU/mL), LV-NC group (negative control, mice received an intra-ovarian injection of 5 µL of LV-NC), and POI group (treated with PBS at the same volume). Except for the control group, the mice in the other groups underwent POI model induction prior to the intervention. One week after the intervention, the mice were sacrificed. The harvested ovarian tissues were fixed with 4% paraformaldehyde, sectioned at 5 μm thickness, and stained with hematoxylin and eosin (H&E). Follicular counting was performed under a light microscope. Granulosa cells were isolated as previously described [28].

### Sample size justification

The sample size of six mice per group was determined based on previous studies demonstrating significant effects in similar murine POI models and ovarian intervention studies using comparable group sizes [29, 30]. This sample size is widely accepted in the field for achieving sufficient statistical power to detect biologically relevant differences in ovarian endpoints under these experimental conditions while adhering to the principle of reduction in animal use.

### Enzyme-linked immunosorbent assay (ELISA)

Blood samples were collected from the abdominal aorta of mice in each group, centrifuged at 3,500 rpm for 15 min to obtain the supernatant. The concentrations of estradiol (E2), anti-Müllerian hormone (AMH), and follicle-stimulating hormone (FSH) were measured using ELISA kits (Maiman, Jiangsu, China) according to the manufacturer’s instructions.

### Flow cytometry detection

Granulosa cells in each group were treated with 200 µL of annexin V binding buffer (Invitrogen) and 5 µL of FITC-conjugated annexin V (Invitrogen), followed by incubation in the dark at room temperature for 15 min. Subsequently, 2 µL of propidium iodide (PI) staining solution (Invitrogen) was added, and the cells were further incubated for 5 min under the same conditions. Fluorescence signals corresponding to FITC and PI were detected using the FL1 and FL2 channels of the flow cytometer, respectively, and the cell populations were quantitatively analyzed.

### RT-qPCR analysis

Total RNA was extracted from granulosa cells and ovarian tissues using TRIzol reagent. cDNA was obtained from the RNA using a reverse transcription kit (Beyotime, Shanghai, China). RT-qPCR was performed using a 7700 Sequence Detection System (Applied Biosystems). The mRNA quantification was performed using the 2^−ΔΔCt^ method. The primer sequences used for PCR analysis are listed in Table S1.

### Statistical analysis

Statistical analyses and graphs were produced using GraphPad Prism (GraphPad Software, San Diego, CA, USA). The t-test was used to compare the differences between two groups. A *P* < 0.05 was considered statistically significant.

## Results

### Identification of mitochondria and PANoptosis-related DEGs

**Fig. 1:**
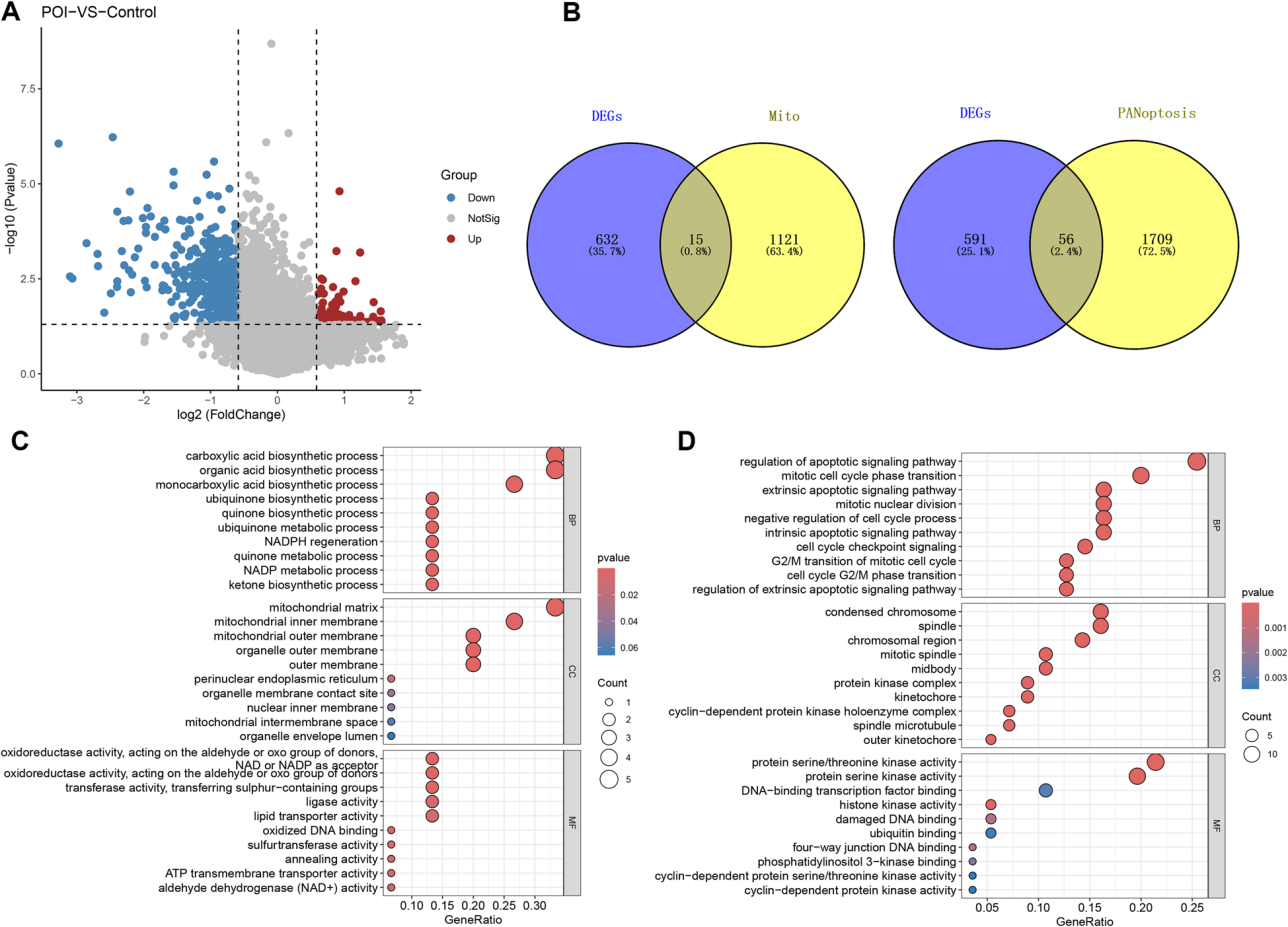
Mitochondria- and PANoptosis-related DEGs identification in POI. **A** Volcano plot of differentially expressed genes; **B** Venn diagram of mitochondria- and PANoptosis-DEGs; **C** Bubble chart of the GO function enrichment results of mitochondria-DEGs; **D** Bubble chart of the GO function enrichment results of PANoptosis-DEGs

Based on the GSE201276 dataset, a total 647 genes were identified as differentially expressed in the POI group compared to the control group, of which 94 were upregulated and 553 were downregulated (Fig. 1A). Subsequently, 1,136 mitochondria-related genes were retrieved from the Human MitoCarta3.0 database and 1,765 PANoptosis-related genes were obtained from previous studies. Using Venn diagram analysis, 15 mitochondrial-DEGs and 56 PANoptosis-DEGs were identified (Fig. 1B).

To explore the biological functions of the mitochondrial and PANoptosis-related DEGs, we conducted GO enrichment analysis. Results suggested that mitochondrial-DEGs were closely related to biosynthesis-related processes, such as carboxylic acid biosynthesis (Fig. 1C). PANoptosis-related DEGs were significantly enriched in apoptosis-related signaling pathways and cell cycle-related biological processes (BP) (Fig. 1D).

### Mitochondria-related biomarkers with machine learning

**Fig. 2:**
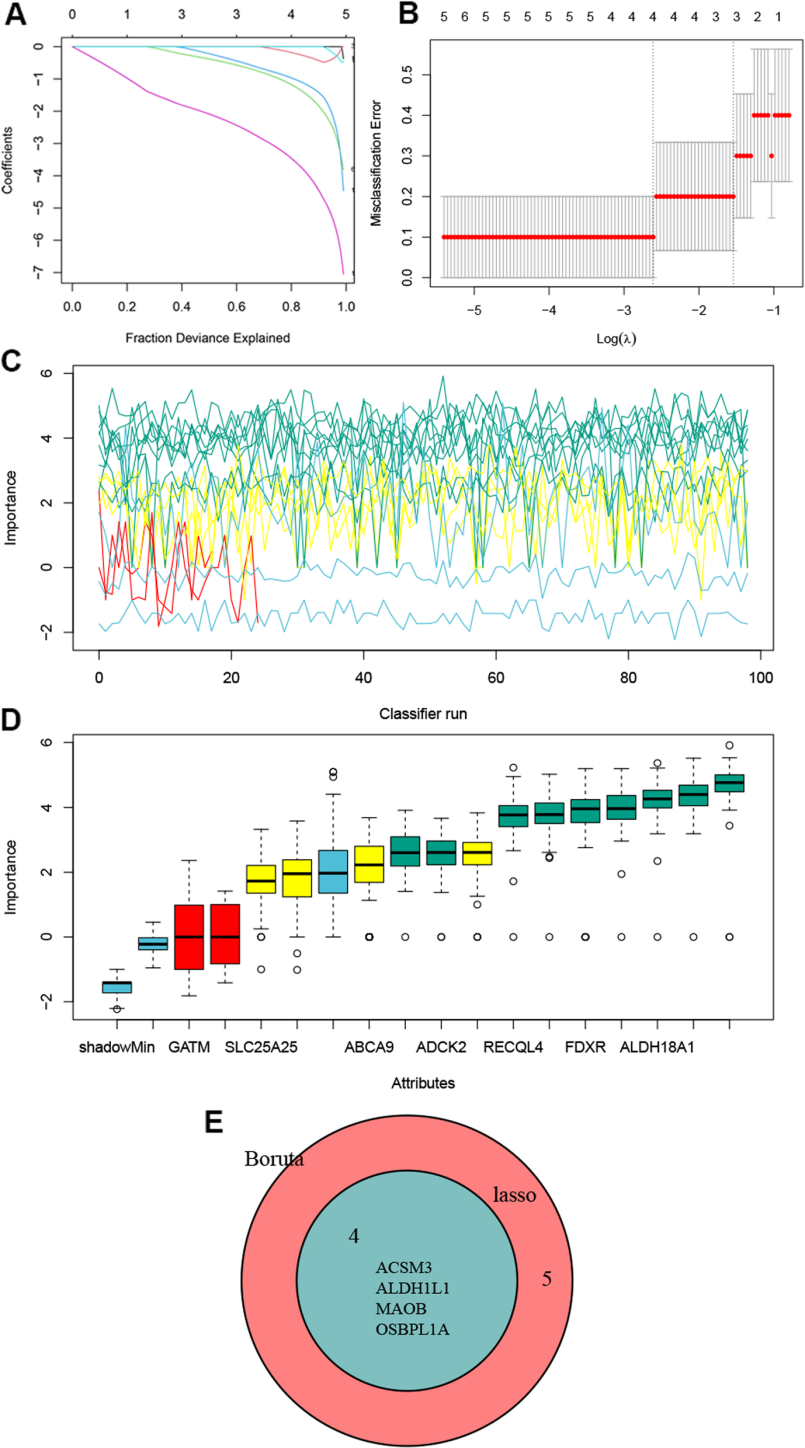
Machine learning methods for feature genes screening of mitochondria-DEGs. **A**, LASSO coefficient profiles. **B**, Identification of the optimal penalization coefficient (lambda) in the Lasso regression and the minimum absolute contraction criterion. **C**, Importance evolution during Boruta run. The green line‌ corresponds to confirmed features, ‌the red line‌ to rejected features, ‌the yellow line‌ to features pending determination, and ‌three blue lines‌ represent the importance of ‌minimum, average, and maximum shaded features‌ respectively. **D**, Boruta result plot for feature importance. Blue boxplot indicates minimal, average and maximum feature importance of a shadow attribute. Red and green boxplots represent feature rejected and confirmed, respectively. Yellow means the importance of confirmed features. **E**, Venn diagram of feature genes identified by two machine learning methods

To mine the key genes from the 15 mitochondrial-DEGs, two machine learning methods were applied. Based on the expression values of the DEGs and sample grouping information (normal and POI), LASSO logistic regression identified four feature genes (*ACSM3*, *ALDH1L1*, *MAOB*, and *OSBPL1A*) (Fig. 2A and B). Nine feature genes were identified using the Boruta model (Fig. 2C and D). After intersection of the feature genes based on the two machine learning methods, four biomarker genes (*ACSM3*, *ALDH1L1*, *MAOB*, and *OSBPL1A*) were identified (Fig. 2E).

### PANoptosis-related biomarkers with machine learning

**Fig. 3:**
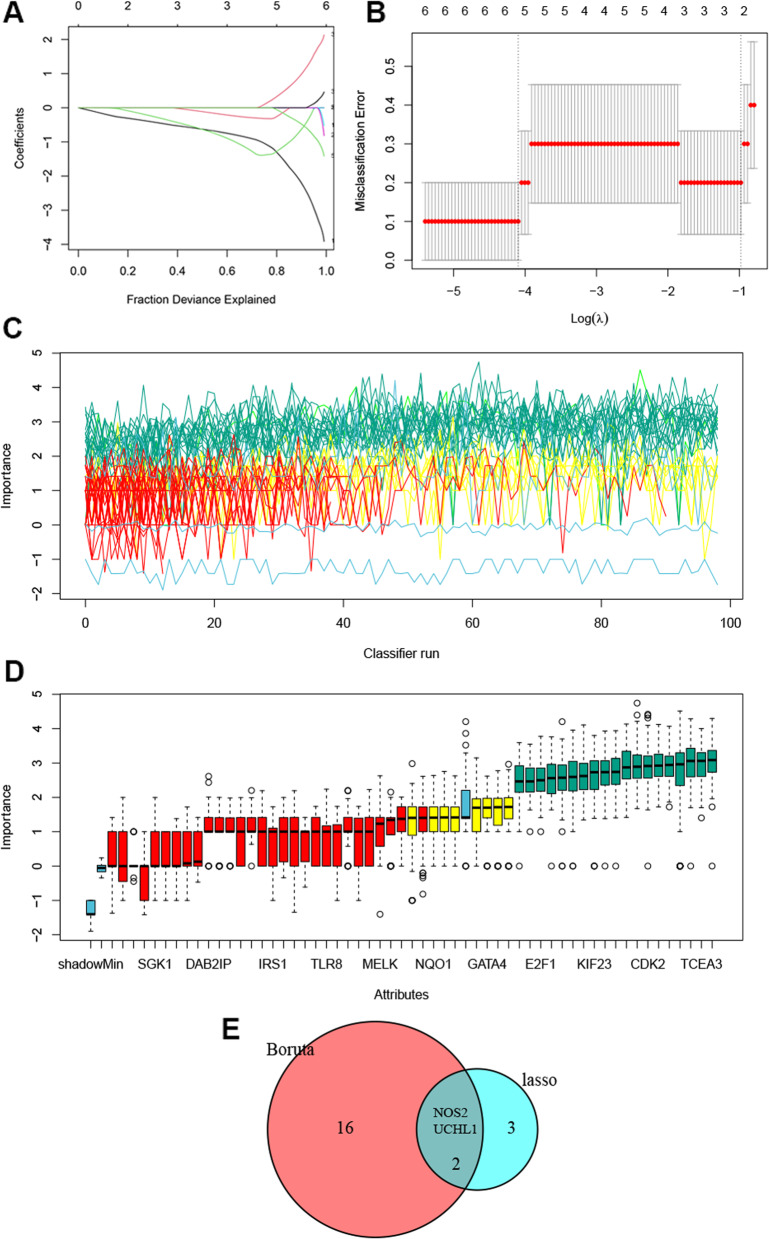
Machine learning methods for feature genes screening of PANoptosis-DEGs. **A,** LASSO coefficient profiles. **B**, Identification of the optimal penalization coefficient (lambda) in the Lasso regression and the minimum absolute contraction criterion. **C**, Importance evolution during Boruta run. Green lines correspond to confirmed attributes, red to rejected ones and blue to respectively minimal, average and maximal shadow attribute importance. **D**, Boruta result plot for feature importance. Blue boxplot indicates minimal, average and maximum feature importance of a shadow attribute. Red and green boxplots represent feature rejected and confirmed, respectively. Yellow means the importance of confirmed features. **E**, Venn diagram of feature genes identified by two machine learning methods

Based on 56 PANoptosis-DEGs, LASSO and Boruta model identified five and 19 key genes associated with the diagnosis of POI, respectively (Fig. 3A-D). Subsequently, the intersection analysis identified two biomarker genes, *NOS2* and *UCHL1* (Fig. 3E).

### Biomarker validation by gene expression and nomogram construction

**Fig. 4:**
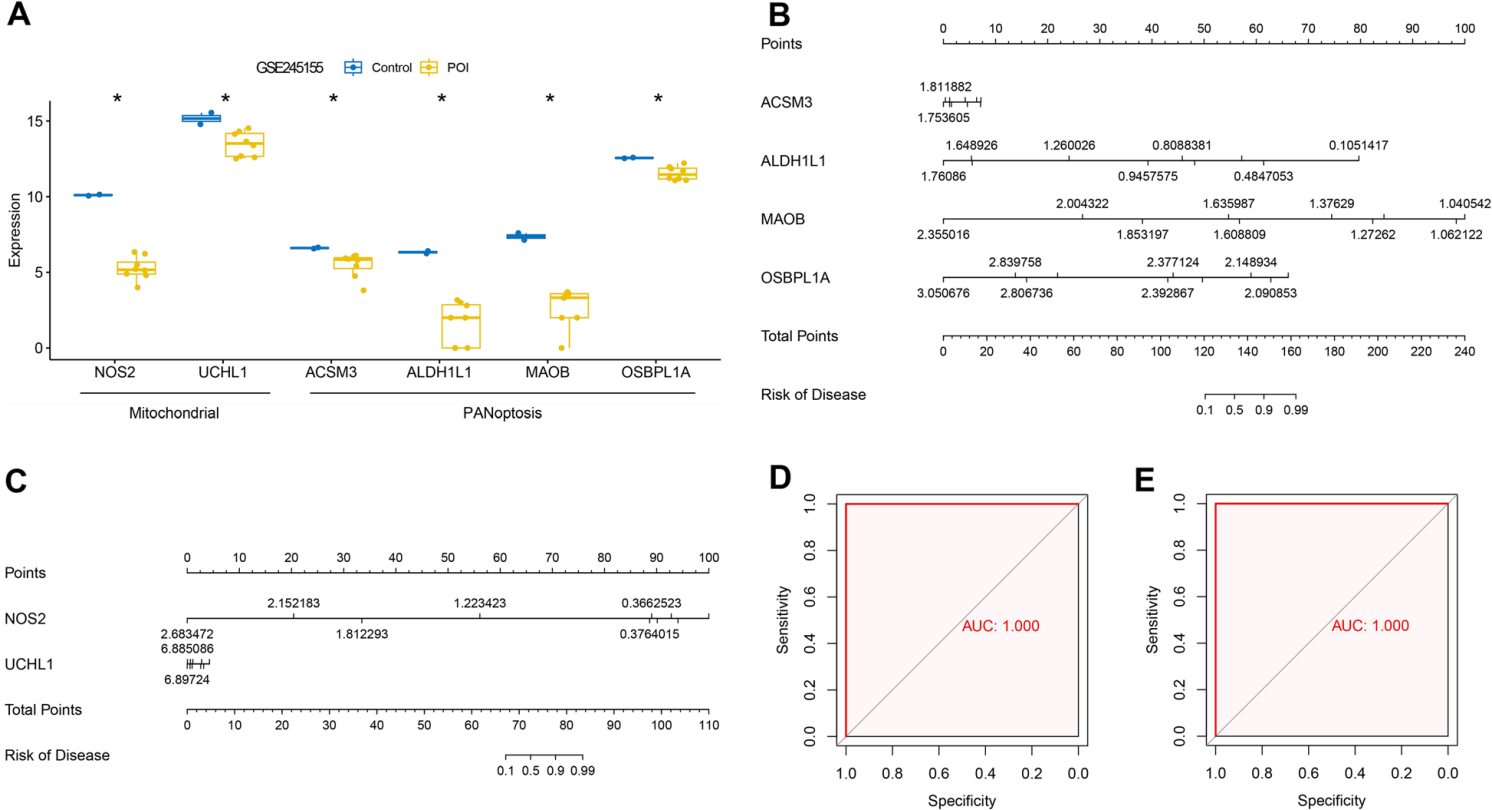
Expression validation and nomogram model construction for feature genes. **A**, Expression validation of feature genes in validation database. **B**, nomogram model construction of mitochondria-feature genes. **C**, nomogram model construction of PANoptosis-related feature genes. **D**, ROC curve was used to analyze the diagnostic value of mitochondrial characteristic genes. **E**, ROC curve was used to analyze the diagnostic value of PANoptosis-related feature genes

Six candidate biomarkers related to mitochondria or PANoptosis were screened: *NOS2*, *UCHL1*, *ACSM3*, *ALDH1L1*, *MAOB*, and *OSBPL1A*. The expression profiles of the biomarker genes were extracted from the GSE245155 validation dataset. As depicted in Fig. 4A, the six biomarkers were downregulated in the POI group compared with the control group, which was consistent with the expression profiles in the training dataset. Nomogram, a practical tool for visualizing multi-gene risk scores, graphically predicts individual disease risk by integrating key gene expression levels. Mitochondrial and PANoptosis-related biomarker genes were incorporated into the nomogram model (Fig. 4B and C). ROC analysis showed that the AUC values of mitochondrial and PANoptosis-related feature genes were both 1 (Figs. 4D and E).

### Mitochondria and PANoptosis phenotypic score and their correlation with immune cells

**Fig. 5:**
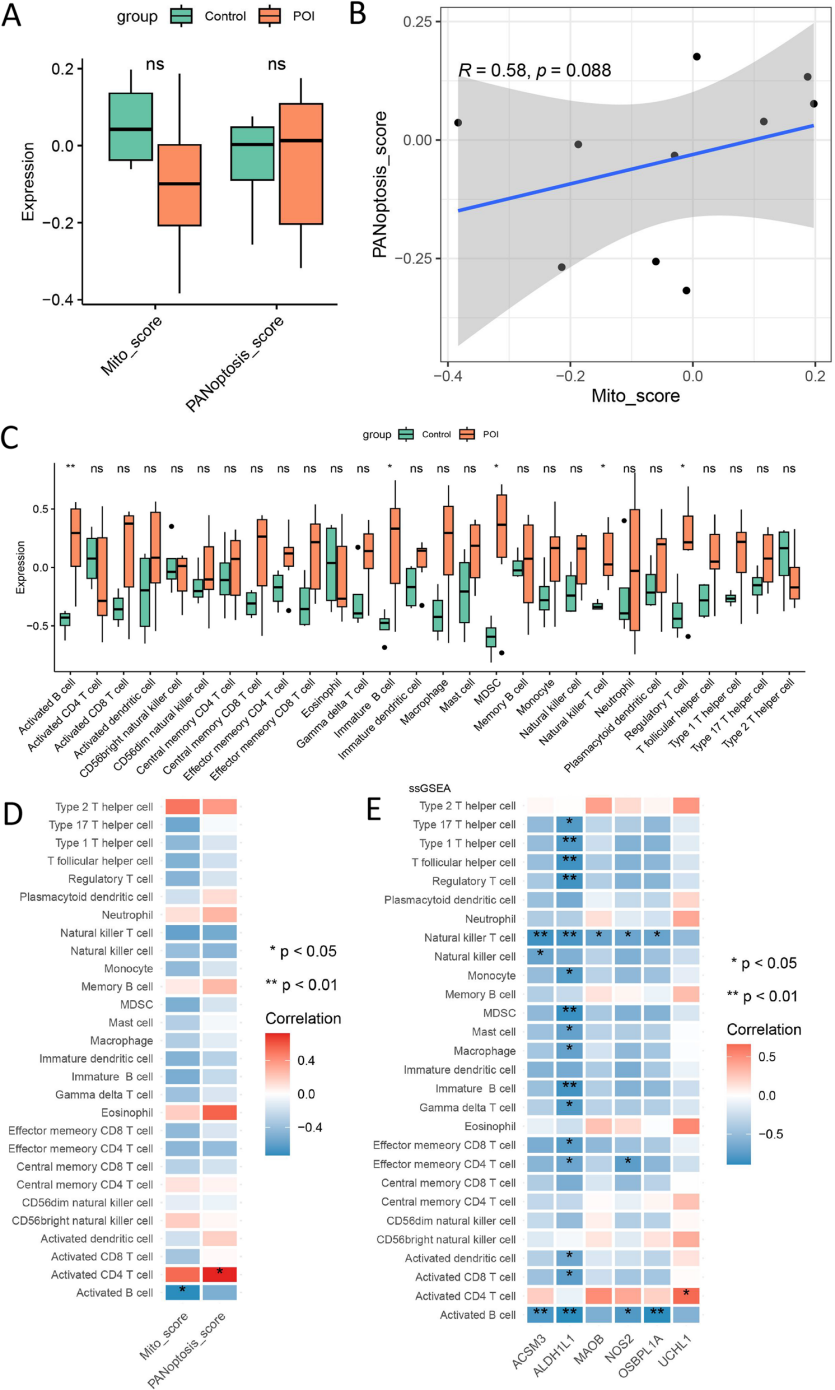
Phenotype scores of mitochondria and PANoptosis and immune infiltration analysis. **A**, difference of the mitochondria and panapoptosis score between POI and control group. **B**, correlation between mitochondria and panapoptosis score. **C**, Box plot of the immune cell infiltration calculated by ssGSEA. **D**, correlation of immune cell infiltration with mitochondria and PANoptosis scores. **E**, Heatmap of the correlation of immune cell infiltration with feature genes

To quantify the mitochondrial and PANoptosis phenotypes in individual patients with POI, we used a scoring system based on mitochondrial and PANoptosis phenotype-relevant genes using the GSVA algorithm. The box plot analysis revealed no significant difference in mitochondrial and PANoptosis scores between POI patients and the control group (Fig. 5A). Spearman’s correlation analysis a positive association between the PANoptosis score and the mitochondrial score (*R* = 0.58, *p* = 0.088) (Fig. 5B). Furthermore, the abundance of 28 types of immune cells was estimated using ssGSEA. Five types of immune cells displayed distinct abundance between the POI and control groups: activated B cells, immature B cells, MDSC, natural killer T cells, and regulatory T cells (Fig. 5C). Activated B cells were negatively correlated with the mitochondrial score, while activated CD4 + T cells were positively correlated with PANoptosis scores (Fig. 5D). Moreover, we observed a correlation between biomarker genes and immune cell populations. Notably, *ALDH1L1* was negatively correlated with most immune cell populations (Fig. 5E).

### Transcriptional regulation network and candidate drugs

**Fig. 6:**
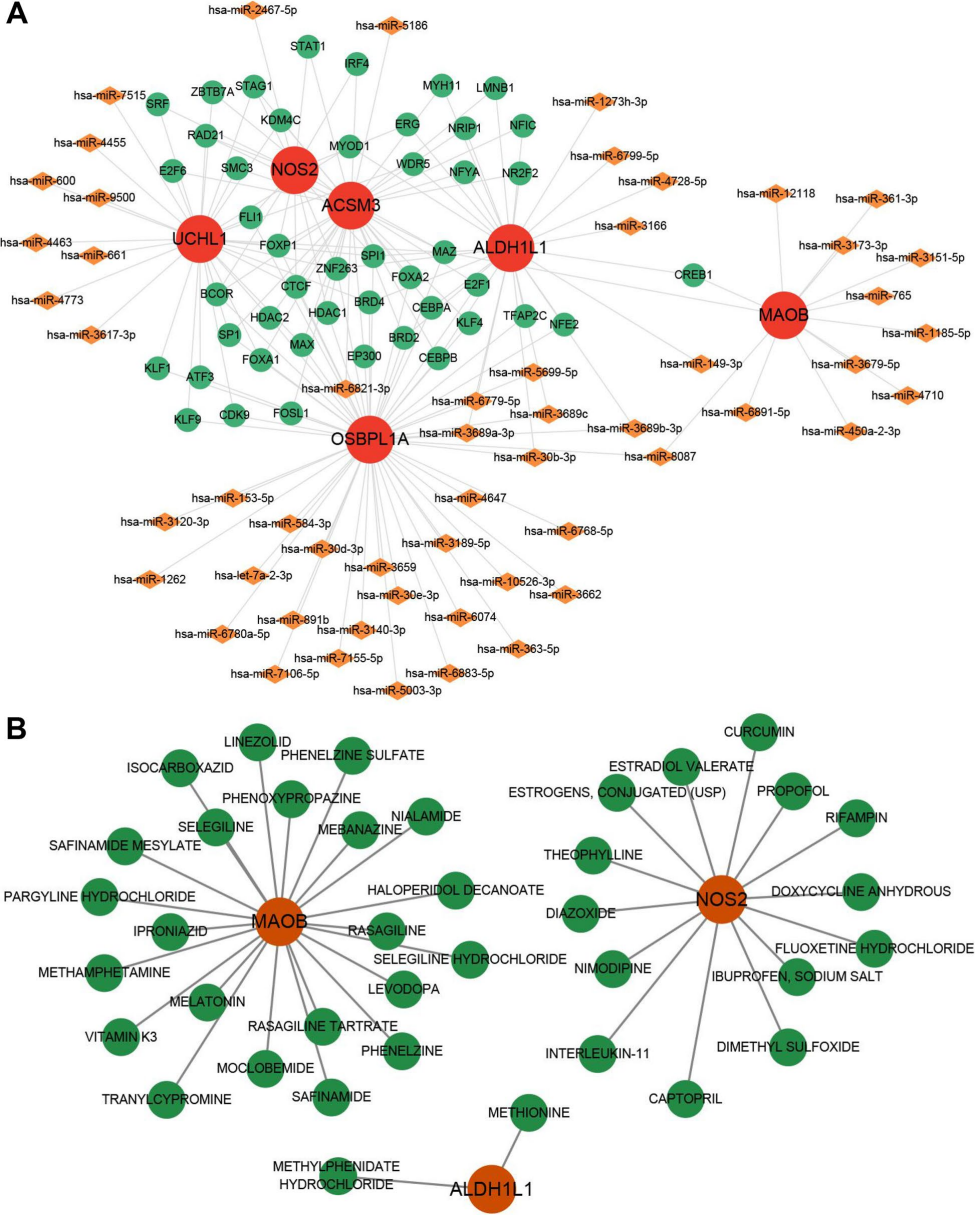
Transcriptional regulation network and drug-gene network. **A**, transcriptional regulatory network. Red represents the feature gene, green represents TF, and orange represents miRNA. **B**, green indicates drugs and red means feature genes

To explore the regulatory mechanisms of these biomarker genes, we predicted the TFs and miRNAs that regulate the biomarker genes and obtained a total of 112 TFs. Further screening revealed that 46 TFs had regulatory relationships with at least two biomarker genes. Ultimately, a total of 181 pairs of TF-target or miRNA-target relationships were obtained, including 46 TFs, 55 miRNAs, and 6 genes (Fig. 6A). Furthermore, using the DGIdb database, we identified small-molecule drugs related to biomarker genes. A drug-target interaction network was constructed, which comprised three biomarker genes, 38 drugs, and 38 interactions (Fig. 6B). *ALDH1L1* showed interaction with methylphenidate hydrochloride and methionine.

### ‌LV-NOS2 increases ovarian follicle quantity‌

**Fig. 7:**
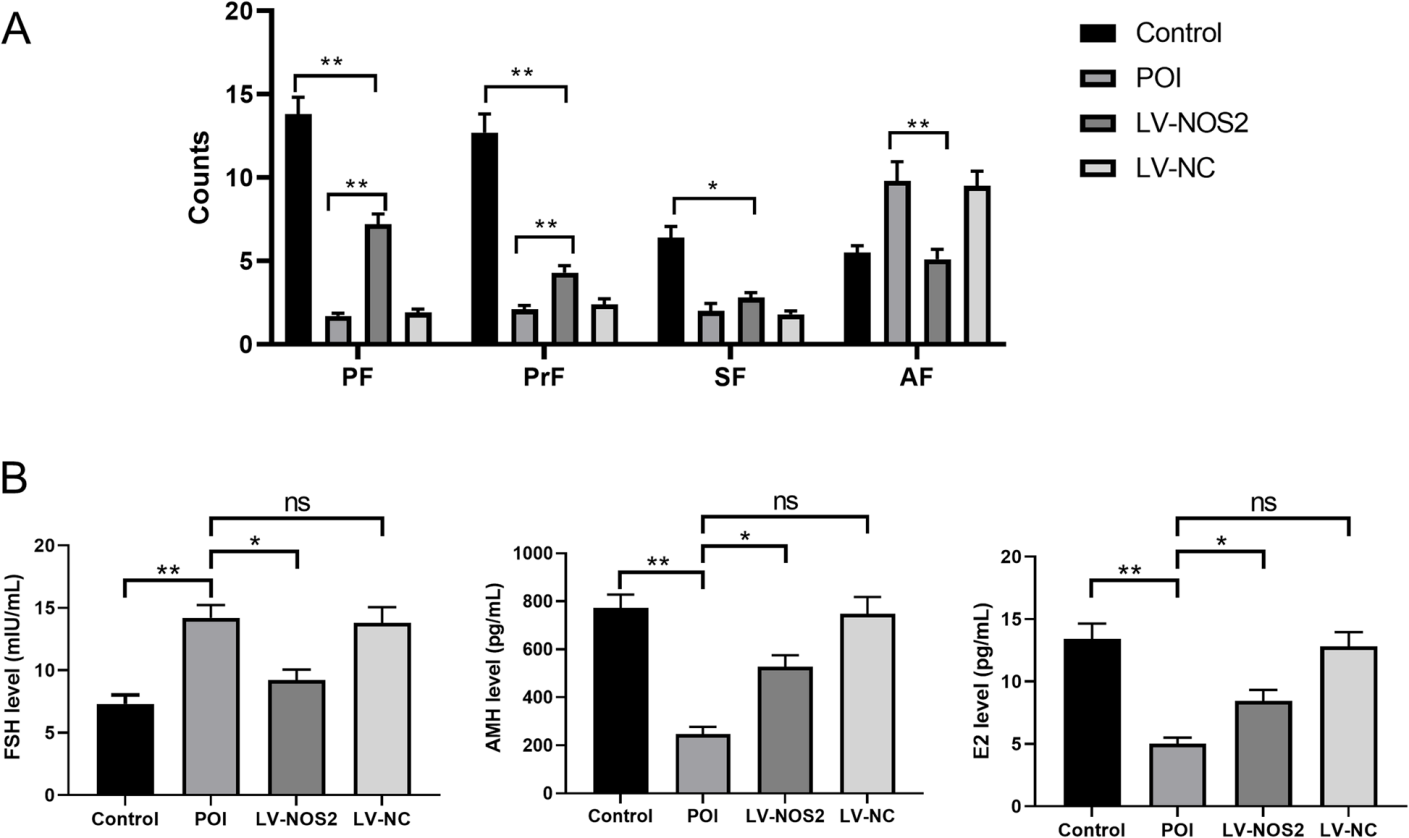
‌Follicle Counting and Serum Levels of E2, FSH, and AMH in Mice‌. ‌**A**, Follicle Counting.‌ ‌PF:‌Primordial follicle; ‌PrF:‌Primary follicles; ‌SF:‌Secondary follicles; ‌AF:‌Atretic follicles. ‌**B**, ELISA Detection of FSH, AMH, and E2 Levels in Mouse Serum.‌ ‌*, *P*< 0.05; ‌‌**, *P* < 0.01;‌ ‌ns, not significant

In the POI animal model, the Control group exhibited follicles at all developmental stages in the ovaries. In contrast, the POI group showed a reduction in the number of growing follicles at all stages and an increase in atretic follicles (AFs). Compared with the POI and LV-NC groups, the LV-NOS2 group exhibited ‌increased numbers of primordial follicles (PFs) and primary follicles (PrFs)‌ along with ‌reduced AFs (Fig. 7A)‌. These findings indicate that LV-NOS2 has the potential to ‌enhance ovarian follicle quantity‌.

### LV-NOS2 reduces FSH levels while increasing AMH and E2 levels

We measured the expression levels of FSH, AMH, and E2 in serum using ELISA. Compared to the Control group, the POI group exhibited elevated FSH levels and significantly reduced levels of AMH and E2. In contrast, LV-NOS2 intervention significantly decreased FSH levels while markedly increasing AMH and E2 levels compared to the POI and LV-NC groups (Fig. 7B). These results demonstrate that LV-NOS2 effectively ameliorates ovarian dysfunction by modulating FSH, AMH, and E2 levels, thereby alleviating the pathological state of POI.

### Expression validation of the biomarker genes

**Fig. 8:**
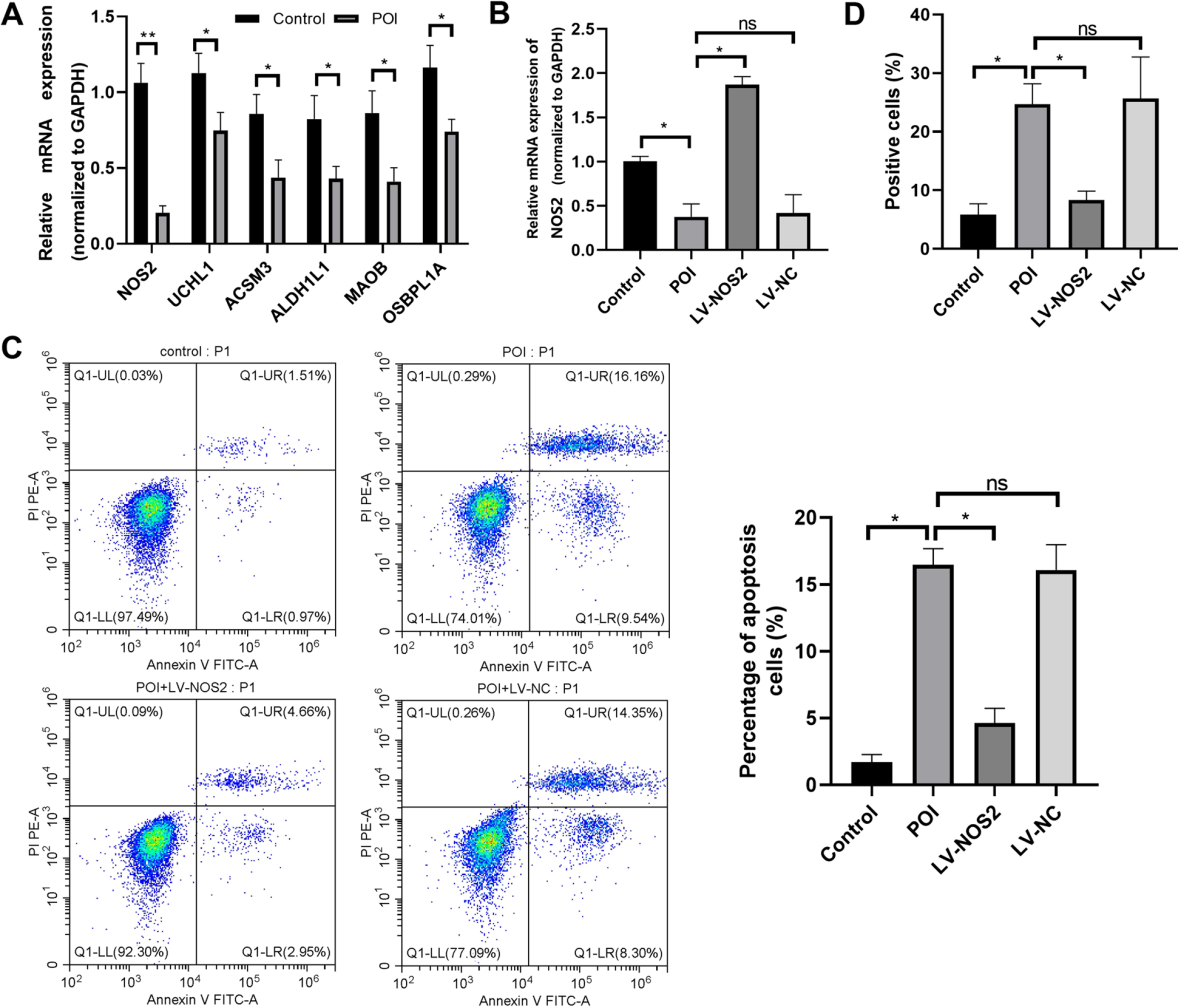
Biomarker gene validation in clinical samples and mice model. **A**, Relative expression of feature genes normalized to *GAPDH* in human granulosa cells (RT-qPCR). **B**, *NOS2* expression level normalized to *GAPDH* in mouse ovary tissues. **C**, flow cytometry detection of cell apoptosis. **D**, flow cytometry detection of ROC generation. ‌*, *P* < 0.05; ‌‌**, *P* < 0.01;‌ ‌ns, not significant

The differential expressions of biomarker genes were validated in human granulosa cells. RT-qPCR analysis showed that the mRNA levels of *NOS2*, *UCHL1*(PGP9.5), *ACSM3*, *ALDH1L1*, *MAOB*, and *OSBPL1A* were significantly lower in POI group, compared with controls (all *P* < 0.05, Fig. 8 A).

#### NOS2 overexpression attenuated PANoptosis in mice POI models

Considering that *NOS2* exhibited the most significant differential expression among the biomarker genes, we explored the role of *NOS2* in POI pathology. RT-qPCR analysis suggested that, compared with the control group, *NOS2* was significantly downregulated in granulosa cells of POI models, but remarkably increased in POI mice injected with LV-NOS2, indicating a high transfection efficiency (all *P* < 0.05, Fig. 8B). Flow cytometric analysis showed that the apoptotic rates of granulosa cells in ovarian tissues of POI mice were markedly elevated, which was abolished by LV-NOS2 injection (Fig. 8 C). Parallelly, the ROS levels in the LV-NOS2 group were significantly decreased in LV-NOS2 group, compared the PIO group (Fig. 8D). Collectively, *NOS2* overexpression significantly suppressed PANoptosis by reducing ROS production. 

## Discussion

POI is a severe health concern that seriously affects the physical and mental health of patients. Although the incidence of POI is relatively low, at 1% in women aged < 40 years and 0.1% in those aged < 30 years [31], it is associated with serious consequences such as menstrual disorders, psychological distress, and even infertility. The current treatment option for infertility is only ovum donation in women with ovarian dysfunction [32]. The etiology of POI is unknown in >50% of patients. In some cases, POI is asymptomatic, and only patients with a family history of POI may benefit from diagnostic testing. Currently, there is no effective treatment for POI. Therefore, biomarkers for the diagnosis and treatment of POI are urgently needed.

In biomarker discovery, machine learning algorithms have been widely applied, providing more accurate predictions. Boruta, a novel machine learning method based on random forest, reduces errors in feature selection and elicits superior advantages in terms of the accuracy, stability, and data classification of feature selection [33–35]. However, application of the Boruta method to gene expression datasets of POI is rare. In this study, four mitochondria-related biomarkers (*ACSM3*, *ALDH1L1*, *MAOB*, and *OSBPL1A*) and two PANoptosis-related biomarkers (*NOS2* and *UCHL1*) were identified using the Boruta algorithm in combination with LASSO. Based on bioinformatics analysis, all biomarker genes were significantly downregulated in patients with POI in the training and validation datasets.

We extracted human granulosa cells and constructed POI mice models for biomarker gene validation. The differential expression profiles of biomarker genes in POI were consistent with those of the bioinformatics analysis. As mentioned in previous studies, mitochondria play an influential role in regulating oogenesis, oocyte maturation, fertilization, and embryonic development [7, 36] and participate in ROS production, ATP generation, and cell death regulation [37]. Granulosa cells are the basic units of ovaries [38]. Mitochondrial dysfunction contributes to energy metabolism disorders, granulosa cell apoptosis, and subsequent POI development [39]. Considering the critical role of granulosa cell disorders in the pathology of POI, we investigated the gene expression profile of granulosa cells in conjunction with mitochondria-related and PANoptosis-related genes. A total of 15 mitochondrial-DEGs and 56 PANoptosis-DEGs were screened, among which four mitochondria-DEGs (*ACSM3*, *ALDH1L1*, *MAOB*, and *OSBPL1A*) and two PANoptosis-DEGs (*NOS2* and *UCHL1*) were filtered using machine learning methods. Although little is known about the relationship between mitochondria and PANoptosis in POI, mitochondrial scores showed a significant positive association with PANoptosis in the present study.

*ACSM3* is an acyl coenzyme A synthase, located on the outer membrane of mitochondria, which is involved in fatty acid metabolism by interacting with medium-chain fatty acids. Reduced *ACSM3* levels are associated with fatty acid accumulation [40]. A previous study reported that the dysregulation of fatty acids in the ovary contributes to the development of POI [41]. In addition, ACSM3 also has significant value in ovarian cancer. Its low expression can promote cell proliferation, migration and invasion and inhibit cell apoptosis by activating JAK/STAT3 signaling pathway [42]. *MAOB* is elevated in the uterus during the peri-implantation period, which is crucial for maintaining endometrial receptivity [43]. Additionally, loss of *UCHL1* is associated with decreased oocyte quality and follicular development, leading to female infertility [44]. Lack of estradiol production in granulosa cells impairs follicular development and leads to failure to ovulate. Overexpression of UCHL1 promotes estradiol synthesis in granulosa cells [45]. These results indicated the potential role of biomarker genes in POI. This study found that compared with the control group, the expressions of ACSM3 and UCHL1 in the POI group were significantly decreased. In the subsequent research, we will construct the ACSM3 and UCHL1 lentiviral vectors and further conduct functional verification of these two genes in vivo.

The immune system plays an important role in POI, and cellular immune dysfunction is related to idiopathic POI [46]. Immunoanalysis revealed that five types of immune cells showed significantly different abundance levels between the POI and control groups: activated B cells, immature B cells, MDSC, natural killer T cells, and regulatory T cells. Among these, activated B cells demonstrated a negative correlation with mitochondrial scores, while activated CD4 + T cells showed a positive correlation with PANoptosis scores. Mitochondria are involved in the differentiation and activation processes of immune cells; in pro-inflammatory cells (e.g., activated monocytes, T cells, and B cells), energy is produced by increasing glycolysis [47]. Mitochondrial loss and dysfunction drive T cell depletion, and mitochondria-enhanced CD8 + T cells mediate superior antitumor responses and prolong animal survival [48]. PANoptosis-mediated death of tumor and immune cells regulates the tumor microenvironment (TME), impacting immunotherapy efficacy [49]. Additionally, PANoptosis participates in thyroid cancer immune evasion by modulating macrophages, CD4^+^T cells, activated T cells, B cells, and TNF signaling pathways [50]. Thus, immune cells are inextricably linked to mitochondrial and PANoptosis dysregulation.

The TFs-miRNAs regulatory network is a complex interaction system; in which key TFs and miRNAs jointly regulate gene expression and affect cell function and disease progression ([51]. In this study, the key regulatory factors included *NOS2*, *UCHL1*, *OSBP1A*, *ACSM3*, *ALDH1L1* and *MAOB*. They regulate various functions and metabolic processes of cells through complex interaction networks. *NOS2*, as an important regulatory factor, has been found to be the target of *miR-493-5p* and is involved in the inflammatory response during bacterial infection ([52]. *MAOB* is also one of the key regulatory factors. Through a complex transcriptional regulatory network, it interacts with multiple ‌miRNAs‌ and may play a core role in the course of specific diseases. A previous study found that *miR-522* accelerates the progression of endometrial cancer by inhibiting *MAOB *[53]. In conclusion, these key regulatory factors jointly influence the occurrence and development of diseases through a complex miRNA regulatory network. Moreover, we also identified small molecule drugs related to biomarker genes. For example, *NOS2* interacts with curcumin. Curcumin is a natural polyphenolic compound extracted from turmeric and has antioxidant, anti-inflammatory, anti-tumor, and anti-cancer activities [54]. Curcumin, as a new potential drug, can promote the development of human small follicles and is used to treat infertility [55]. Previous studies have found that curcumin alleviates liver injury induced by heat stroke in a dose-dependent manner by down-regulating NF-κB, *NOS2*, and ICAM-1 [56]. In the pathological mechanism of POI, local inflammation of the ovary and oxidative stress are important factors accelerating follicular exhaustion [57]. Therefore, we speculate that curcumin alleviates ovarian microenvironment damage and delays follicular degeneration by targeting *NOS2*. However, the drug-gene interaction network includes drugs that are not related to the clinical practice of POI. Therefore, the efficacy and mechanism of these drugs in POI require further experimental verification.

Given the significant differences in *NOS2* among our biomarkers, we further investigated the role of *NOS2* in POI. Our results suggested that *NOS2* was downregulated in granulosa cells of patients with POI and mice models. *NOS2* overexpression significantly reduced the death rate of granulosa cells in the ovarian tissues of POI mice, in parallel with a decline in ROS production. Previous studies have demonstrated the critical role of nitric oxide (NO) in human ovarian physiology. *NOS2* is reported to be downregulated in rat granulosa cells, which is associated with a caspase-mediated cell apoptosis [58]. Additionally, the decreased expression of *NOS2* induces an increase in apoptotic cell death in granulosa cells in a bovine model [59]. The results of these previous studies are consistent with our findings. Therefore, targeting *NOS2* may be a novel treatment option for POI.

However, there are several limitations of this study that need to be noted. Firstly, the control sample size of GSE245155 is small, which may affect the statistical power and general applicability of the research results. Secondly, this study only conducted functional validation on one biomarker (*NOS2*). Therefore, in the next step, we will incorporate a larger sample dataset, increase financial support, further verify the results of this study and conduct functional experiments on other genes.

## Conclusions

In conclusion, we identified six mitochondrial and PANoptosis-related biomarkers. Biomarkers may play key roles in POI pathology. *NOS2* was downregulated in POI and its overexpression reduced granulosa cell death and ROS production. The biomarkers may be candidates for the diagnosis and treatment of POI.

## Supplementary Information

Supplementary Material 1.

## Data Availability

The datasets used and/or analysed during the current study are available from the corresponding author on reasonable request.

## Abbreviations

POI: Premature ovarian insufficiency
GEO: Gene expression omnibus
DEGs: Differential expressed genes
ROS: Reactive oxygen species
TFs: Transcription factors
miRNAs: microRNAs
LV-NOS2: Lentivirus vector carrying NOS2 gene sequence
PI: Propidium iodide
NOS2: Nitric oxide synthase
CTX: Cyclophosphamide
ELISA: Enzyme-linked immunosorbent assay
E2: Estradiol
AMH: Anti-Müllerian hormone
FSH: Follicle-stimulating hormone
BP: Biological processes
AFs: Atretic follicles
PFs: Primordial follicles
PrFs: Primary follicles
TME: Tumor microenvironment
NO: Nitric oxide
