# Supplementary material for: Unveiling mitochondrial and PANoptosis-related biomarkers for premature ovarian insufficiency
Source: J Ovarian Res. 2025 Dec 22;18:297. doi: 10.1186/s13048-025-01839-4 (PMC12723934; doi:10.1186/s13048-025-01839-4)
Supplement: Supplementary file 1 — Supplementary Material 1. [file 13048_2025_1839_MOESM1_ESM.docx]

**Table S1 Primer sequences for RT-qPCR analysis**

| Gene  symbol | Primer sequence  (5’ - 3’) | Accession numbers‌  (NCBI) |
| --- | --- | --- |
| ACSM3 | F: AAGGTTCAGGGCTGCTCTTC | [NM_005622.4](https://www.ncbi.nlm.nih.gov/entrez/viewer.fcgi?db=nucleotide&id=1519244062) |
|  | R: AGCATCTTCCTGGTGACACG |  |
| ALDH1L1 | F: CCCCTGGAACTATCCCCTGA | XM_024453325.2 |
|  | R: TGGGAATGCCGGCCTTTAAT |  |
| MAOB | F: GGCGGCATCTCAGGTATGG | NM_000898.5 |
|  | R: GGTCTCCAATCCTAGCTCCTTG |  |
| OSBPL1A | F: TCCGAAGAAAAAGACTGTGGTG | XM_054318171.1 |
|  | R: CAGTTAGGCGCTGTAGGAAGC |  |
| NOS2 | F: CGCATGACCTTGGTGTTTGG | NM_000625.4 |
|  | R: CATAGACCTTGGGCTTGCCA |  |
| UCHL1 | F: AGGGACAGGAAGTTAGCCCTA | NM_004181.5 |
|  | R: AGCTTCTCCGTTTCAGACAGA |  |
| GAPDH | F: GGGAAACTGTGGCGTGAT | NM_002046.7 |
|  | R: GAGTGGGTGTCGCTGTTGA |  |
